# Missed Registration of Disease Codes for Pediatric Anaphylaxis at the Emergency Department

**DOI:** 10.1155/2019/4198630

**Published:** 2019-08-14

**Authors:** Byungho Choi, Sun Hyu Kim, Hyeji Lee

**Affiliations:** Department of Emergency Medicine, Ulsan University Hospital, University of Ulsan College of Medicine, 877 Bangeojinsunhwando-ro, Dong-gu, Ulsan 44033, Republic of Korea

## Abstract

**Background:**

It is important to register anaphylaxis codes correctly to study the exact prevalence of anaphylaxis. The purpose of this study was to analyze the clinical characteristics and disease codes of inaccurately registered groups in pediatric anaphylaxis patients.

**Methods:**

This study reviewed the medical records of all pediatric patients who presented to the university hospital emergency department over a 5-year period. Study subjects were divided into 2 groups: the accurate group, including those registered under anaphylaxis codes, and the inaccurate coding group, including those registered under other codes.

**Results:**

From a total of 79,676 pediatric patients, 184 (0.23%) had anaphylaxis. Of these, 23 (12.5%) and 161 (87.5%) patients were classified to the accurate and inaccurate coding groups, respectively. Average age, time from symptom onset to emergency department presentation, past history of allergy, and penicillin and cephalosporin as causes of anaphylaxis differed between the 2 groups. Cardiovascular (39.1% vs. 5.6%, *p*=0.001) and respiratory symptoms (65.2% vs. 42.2%, *p*=0.038) manifested more frequently in the accurate group, while gastrointestinal symptoms (68.3% vs. 26.1%, *p*=0.001) were more frequently observed in the inaccurate coding group. Fluid administration (82.6% vs. 28.0%, *p*=0.001), steroid use (60.9% vs. 23.0%, *p*=0.001), and epinephrine use (65.2% vs. 13.0% *p*=0.001) were more common treatments for anaphylaxis in the emergency department in the accurate group. Anaphylaxis patients with cardiovascular symptoms, steroid use, and epinephrine use were more likely to be accurately registered with anaphylaxis disease codes.

**Conclusions:**

In the case of pediatric anaphylaxis, more patients were registered inaccurately under other allergy-related codes and simple symptom codes, rather than under anaphylaxis codes. Therefore, future research on anaphylaxis should consider inaccurately registered anaphylactic patients, as shown in this study.

## 1. Background

Anaphylaxis is a life-threatening systemic allergic reaction typically caused by food, drugs, or insect venom. Anaphylaxis is a condition with variable presentations ranging from mild allergic reaction to major cardiovascular compromise. Due to the variable presentation and sudden nature of anaphylaxis, most patients with anaphylaxis visit the emergency department, and accurate diagnosis and prompt treatment by emergency department physicians are important. Anaphylaxis was defined using criteria from the 2011 World Allergy Organization Guidelines for the Assessment and Management of Anaphylaxis [1]. The prevalence of anaphylaxis has increased in both adults and children worldwide over the past 2 decades [2, 3]. Globally, the prevalence and causes of anaphylaxis have been reported, and the prevalence of anaphylaxis in the general population in the United States has been reported in 1.6% to 5.1%, and 1 of 1,333 (0.1%) to 37 of 6,676 (0.6%) in Europe [4, 5]. Poulos et al. reported a rise in anaphylaxis hospitalization in Australian children aged 0–4 years from 4.1 to 19.7 per 100,000 person-years, especially a 5.5-fold increase in food-triggered reactions [6]. And the frequency of anaphylaxis cases especially visiting the emergency department has risen over the past several years, with one report citing a 58% rise [7].

Although the prevalence of anaphylaxis is increasing and this remains an active research area, in many studies, the study population is limited to patients registered as having anaphylaxis. As a result, patients who met the diagnostic criteria for anaphylaxis but were designated a disease code other than anaphylaxis may have been missed [8]. To study the exact prevalence of anaphylaxis, it is important to register anaphylaxis codes correctly, as anaphylaxis tends to be registered other disease codes instead of the actual anaphylaxis codes [9, 10]. In particular, pediatric patients registered as having angioedema or urticaria are reported to be more likely to be rediagnosed with anaphylaxis than adults [11]. Some studies have attempted to reduce the likelihood of missing patients with anaphylaxis by including allergy-related disease codes such as urticaria, angioedema, and allergy in the study [12–14]. Patients who were registered with other disease codes were still excluded from these studies.

To the best of our knowledge, there are only few reports detailing the number of pediatric anaphylaxis cases that have been registered as diseases other than anaphylaxis. However, the differences in the characteristics of the two groups (accurate and inaccurate coding) are less extensively discussed in the previous reports. Therefore, in this study, we aimed at investigating the frequency and clinical characteristics of anaphylaxis cases registered as other diseases in pediatric patients who visited the emergency department.

## 2. Methods

Subjects included in this study were patients with anaphylaxis aged under 15 years who presented to the pediatric emergency department of a tertiary university hospital over a 5-year period between January 2012 and December 2016. Medical records of all pediatric patients who presented to the emergency department during the study period were reviewed retrospectively, in order to reevaluate if a diagnosis of anaphylaxis was necessary or missing. Primary data sources were the emergency department medical records, including physician and nursing notes, and observation, fluid, and medication charts. Subjects were excluded if they did not fulfill the anaphylaxis diagnostic criteria [1] after medical record review. This study was reviewed and approved by the Institutional Review Board (UUHIRB-2018-04-029). Informed consent was waived by IRB due to the feature of retrospective analysis.

Study subjects were divided into 2 groups: the accurate group, which included codes T78.0, T78.2, T78.2B, T78.2C, T80.5, and T88.6 with the direct specification of anaphylaxis and anaphylactic shock in international classification of diseases 10^th^ version (ICD-10), and the inaccurate coding group, which included those with anaphylaxis registered under all other codes. If a patient had repeated visits, each case was included as a separate presentation [15].

Associated variables were recorded to evaluate the patients' age, sex, personal and family history of allergic diseases, comorbid diseases, causes of anaphylaxis, clinical characteristics, and treatment. We also collected vital signs at emergency department arrival, transportation to the emergency department, elapsed time from exposure to symptom onset, elapsed time from symptom onset to emergency department arrival, and fever. Fever was defined as a temperature greater than 38.0°C. Transportation to the emergency department was classified as either public ambulance, individual transportation, or transfer from another medical facility. Past history of allergy was classified into anaphylaxis, allergic rhinitis, asthma, atopic disease, drugs, and foods. Drugs, insect stings, food, exercise, and idiopathic factors were classified as the possible causes of anaphylaxis. For detailed information regarding the cause of anaphylaxis, drugs were categorized into nonsteroidal anti-inflammatory drugs, penicillin, cephalosporin, acetaminophen, and vaccines. Foods were classified into seafood, wheat, buckwheat, pupa, nuts, egg, kiwi, pork, and cow milk, including powdered milk and milk products. In addition, exercise-induced causes, food-dependent exercise-induced causes, and idiopathic causes were also examined. Clinical manifestations were classified into cutaneous, respiratory, cardiovascular, gastrointestinal, and neurologic symptoms. As persistent gastrointestinal symptoms are not explicitly defined in the diagnostic criteria used, we applied a cutoff period of over 30 minutes. Consciousness and the severity of anaphylactic reactions were also collected. The severity of anaphylactic reactions was graded into nonsevere and severe grades depending on hypoxia, hypotension, and neurologic symptoms. Details of emergency department treatment included oxygen supply, fluid administration, advanced airway management, antihistamine administration, steroid administration, epinephrine administration, bronchodilator administration, and cardiopulmonary resuscitation.

### 2.1. Statistical Analysis

Frequency analyses of the registered codes were performed on both the accurate and inaccurate coding groups. Continuous data were described as the median with interquartile ranges. Univariate comparison analysis was performed using the chi-squared test, Student's *t*-test, Fisher's exact test, and Mann–Whitney *U*-test where appropriate to compare the general characteristics of patients, causes of anaphylaxis, clinical characteristics, and treatments between the 2 groups. To identify factors that were more likely to be registered as anaphylaxis codes, factors that were statistically significant were included in multivariate logistic regression analysis, after correcting for patient sex. Data entry and statistical analysis were performed using IBM SPSS Statistics, version 21.0 (IBM Corp., Armonk, NY, USA). A *p* value of less than 0.05 was considered statistically significant.

## 3. Results

**Figure 1:**
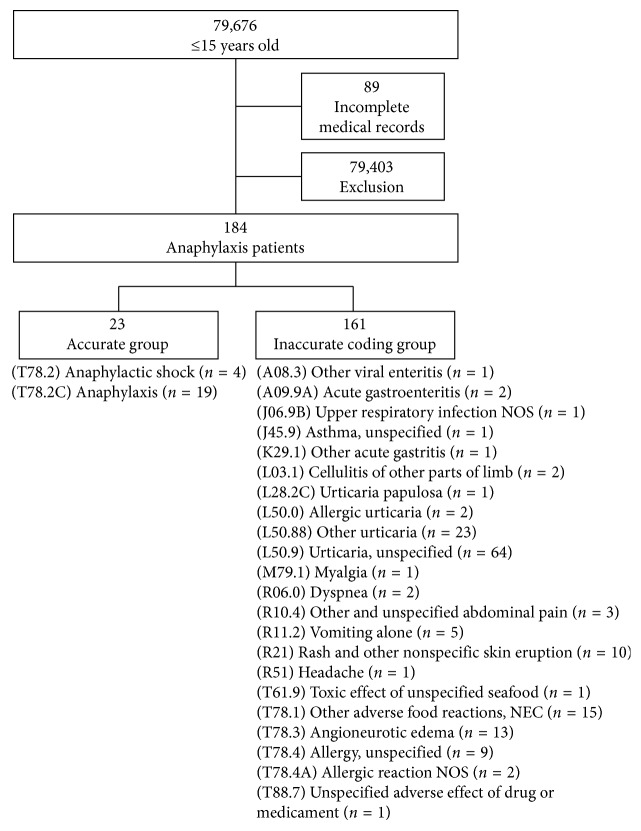
The number of pediatric anaphylaxis cases registered accurately or inaccurately. We excluded patients who did not satisfy anaphylaxis diagnostic criteria.

There were a total of 79,676 pediatric emergency department presentations during the 5-year study period. Whole clinical records were identified and reviewed, excluding cases that had incomplete medical records or did not fulfill the diagnostic criteria for anaphylaxis. Of the 184 cases of anaphylaxis, 23 (12.5%) and 161 (87.5%) were divided into the accurate and inaccurate coding groups, respectively (Figure 1). Of the 23 cases of accurate coding groups, all patients met the diagnostic criteria.

The mean ± standard deviation age of all patients was 4.5 ± 4.7 years; the accurate and inaccurate groups were 7.2 ± 5.1 years and 4.2 ± 4.6 years, respectively. Overall, 56% of anaphylaxis presentations were male and there was no significant difference between the 2 groups. With regard to transportation to the emergency department, public ambulances were used by 21.7% and 3.7% of the accurate and inaccurate groups, respectively; however, overall, there was no significant difference between groups. There was no difference between groups for the time from exposure to symptom onset. However, the time from symptom onset to emergency department arrival was longer in the inaccurate group. Overall, 34.2% of patients had a past history of allergy, and this was 56.5% in the accurate group compared to 31.1% in the inaccurate coding group. The two groups had no differences in comorbid diseases and fever (Table 1).

Foods were the most common cause of anaphylaxis, overall and in both groups. The accurate group had a higher proportion of anaphylaxis caused by drugs than the inaccurate group, with 39.1% and 12.4%, respectively. Analysis of the detailed causes revealed the differences between the 2 groups with respect to penicillin (13.0% vs. 0.0%) and cephalosporin (13.0% vs. 0.6%). Idiopathic cases accounted for 4.3% of the accurate group and 18.0% of the inaccurate coding group (Table 2).

Cutaneous symptoms were the most common symptoms in both groups. The accurate group had more respiratory (65.2% vs. 42.2%), cardiovascular (39.1% vs. 5.6%), and neurologic (13.0% vs. 2.5%) symptoms than the inaccurate coding group, whereas the inaccurate coding group had more gastrointestinal symptoms (68.3%) than the accurate group (26.1%).

There was no difference in systolic or diastolic blood pressure at the time of emergency department arrival between the 2 groups. Severe symptoms were noted in only 1.9% of the inaccurate coding group, but 30.4% of the accurate group had severe symptoms. Altered consciousness occurred in only 8.7% of the accurate group. In the emergency department, treatment with antihistamine was most commonly used. The accurate group had greater fluid administration (82.6% vs. 28.0%), steroid use (60.9% vs. 23.0%), and epinephrine use (65.2% vs. 13.0%) than the inaccurate group (Table 3).

The factors that had statistical significance in the univariate comparison analysis were included in the multivariate logistic regression analysis, after adjusting for sex. The results revealed that patients with anaphylaxis with cardiovascular symptoms, fluid administration, and epinephrine use in the emergency department were more likely to be registered with anaphylaxis codes (Table 4).

As in this study, there is a way to review all patients who visit the emergency department, but it can take a lot of manpower and time. In this study, we investigated the frequency of antihistamine, epinephrine, and steroids commonly used in anaphylaxis and allergic diseases among patients rediagnosed with anaphylaxis following an examination of medical records. Our results show that at least 1 of the 3 drugs was administered in 97.8% of cases (Table 5). There were 4 patients who did not receive any medication; of these, 1 patient in the accurate group was transferred after receiving diagnosis and treatment at another facility, and all 3 in the inaccurate coding group were registered as having urticaria and did not require further treatment with these medications due to their good clinical condition. Therefore, to minimize the number of patients missed due to inaccurate coding in a study of anaphylaxis, the patient group that received antihistamine, epinephrine, or steroids, as well as the patients who were registered with allergy-related codes, should be included in the study group.

## 4. Discussion

To our knowledge, this is the first study to investigate all pediatric cases that meet the diagnostic criteria for anaphylaxis but who were registered with other disease codes in the emergency department. Of the 184 patients who were deemed to meet the diagnostic criteria for anaphylaxis in this study, 23 were previously registered with anaphylaxis codes, while 87.5% of patients were registered with other disease codes, rather than anaphylaxis. Using the diagnostic criteria, the diagnosis of anaphylaxis is based on the history and symptoms of allergen exposure to the patient. It is presumed that it may be difficult to accurately take a history and identify symptoms in pediatric patients, because of their age. Of the 161 registered with other disease codes, 64 cases were registered “urticaria, unspecified.” If “urticaria” was specified, or the visually observable skin and mucosal manifestation codes, such as angioneurotic edema, were included, this accounts for 115 patients, which is more than 70% of the inaccurate coding group (Figure 1). Cutaneous symptoms, such as urticaria and angioneurotic edema, have been reported as the most common symptom in most studies, [1, 16–18] and while this is an objective symptom that is easily observable and clearly distinguishable with the naked eye.

On the other hand, in previous studies, authors reported that, in some cases, anaphylaxis patients were registered with simple symptom and sign codes, such as rash, dyspnea, and abdominal pain, as well as allergy-related codes [15]. There were 21 cases (13.0%) of symptom and sign codes used in this study. A further 12 codes related to gastrointestinal diseases, such as enteritis, gastritis, abdominal pain, and vomiting, were also used in this study. In some studies, approximately 50% of pediatric anaphylaxis cases have been reported to involve gastrointestinal symptoms [18–21]. In this study, 63% of patients had gastrointestinal symptoms, and this was higher in the inaccurate coding group (68.3%) than in the accurate group (26.1%) (Table 3). This suggests that even though gastrointestinal symptoms are included in the anaphylaxis diagnostic criteria, physicians are not well aware of this connection and registered the patient with codes related to gastrointestinal disease.

Pediatric anaphylaxis is less likely to have severe symptoms when compared to adults. In the present study, no cardiopulmonary resuscitation was performed. The frequency of severe symptoms was 30.4% in the accurate group and 1.9% in the inaccurate group. Only 2 cases of altered consciousness were confirmed in the accurate group, which is a similar result to previous studies [22, 23].

In the treatment of anaphylaxis, oxygen supply, antihistamines, and bronchodilator use did not differ between the 2 groups, but there was significantly greater fluid administration, steroid use, and epinephrine use in the accurate group. In particular, patients with epinephrine use or fluid administration were 5.98-fold and 4.51-fold more accurately registered with anaphylaxis codes than patients who did not, respectively (Table 4). This was more often seen with cardiovascular and respiratory symptoms, which are considered to be relatively serious. As severe symptoms were also more common in the accurate group, it is assumed that they were treated more aggressively and correctly registered with anaphylaxis codes.

Similar to reports from previous studies, it was more likely for anaphylaxis to be registered with inaccurate codes, which makes it difficult to accurately assess the prevalence of anaphylaxis. In addition, for pediatric patients with anaphylaxis with a long life expectancy, it can lead to missed opportunities to receive appropriate management for future prevention, by avoiding allergens after consultation with an expert to identify the correct allergen. Therefore, through accurate recognition of diagnostic criteria of anaphylaxis and training of emergency department physicians, the correct anaphylaxis codes can be entered accurately. In future studies concerning anaphylaxis, care should be taken not to omit patients with anaphylaxis.

There are some limitations in this study. This study was a retrospective study conducted at a university hospital; the prevalence of anaphylaxis in the emergency department of a research hospital may differ from other locations. Further, depending on the physician, anaphylaxis codes may be registered differently, making it difficult to generalize the findings.

## 5. Conclusions

In this study, the number of cases of pediatric patients with anaphylaxis who visited the emergency department and were inaccurately registered with nonanaphylaxis codes was higher than those registered accurately with anaphylaxis codes. Cardiovascular symptoms, fluid administration, and epinephrine use in the emergency department were more likely to be registered as anaphylaxis codes. If the patient group that received antihistamine, epinephrine, or steroids, as well as the patients who were registered with allergy-related codes were included in the future study of anaphylaxis, anaphylaxis patients who are being missed can be minimized.

## Figures and Tables

**Table 1:** General characteristics of anaphylaxis cases registered accurately or inaccurately.

Variables	Overall (*n* = 184)	Accurate (*n* = 23)	Inaccurate coding (*n* = 161)	*p*
Average age, years	4.5 ± 4.7	7.2 ± 5.1^∗^	4.2 ± 4.6^∗^	0.003^‡^
Sex, male (%)	103 (56.0)	14 (60.9)	89 (55.3)	0.616
Transportation to ED (%)				0.148
Public ambulance	11 (6.0)	5 (21.7)	6 (3.7)	
Other medical facility	10 (5.4)	2 (8.7)	11 (6.8)	
Individual transportation	160 (87.0)	16 (69.6)	144 (89.4)	
Elapsed time from, min				
Exposure to symptom onset	10 (0–30)^†^	10 (3–30)^†^	10 (0–35)^†^	0.827^§^
Symptom onset to ED arrival	120 (60–210)^†^	60 (30–120)^†^	120 (60–232)^†^	0.046^§^
Past history of allergy (%)	63 (34.2)	13 (56.5)	50 (31.1)	0.016
Anaphylaxis	1 (0.5)	1 (4.3)	0 (0.0)	0.125^||^
Asthma	3 (1.6)	0 (0.0)	3 (1.9)	1.000^||^
Allergic rhinitis	8 (4.3)	2 (8.7)	6 (3.7)	0.263^||^
Atopic dermatitis	19 (10.3)	4 (17.4)	15 (9.3)	0.266^||^
Drug allergy	6 (3.3)	2 (8.7)	4 (2.5)	0.164^||^
Food allergy	33 (17.9)	5 (21.7)	28 (17.4)	0.570^||^
Comorbid diseases (%)	7 (3.8)	1 (4.3)	6 (3.7)	1.000^||^
Fever (%)	18 (9.8)	1 (4.3)	17 (10.6)	0.705^||^

^*∗*^Mean ± standard deviation; ^†^median (interquartile range); ^‡^Student's *t*-test; ^§^Mann–Whitney test; ^||^Fisher's exact test. ED = emergency department.

**Table 2:** Triggers of anaphylaxis cases registered accurately or inaccurately.

Variables	Overall (*n* = 184)	Accurate (*n* = 23)	Inaccurate coding (*n* = 161)	*p*
Drug (%)	29 (15.8)	9 (39.1)	20 (12.4)	0.003^*∗*^
NSAIDs	4 (2.2)	2 (8.7)	2 (1.2)	0.077^*∗*^
Penicillin	3 (1.6)	3 (13.0)	0 (0.0)	0.002^*∗*^
Cephalosporin	4 (2.2)	3 (13.0)	1 (0.6)	0.006^*∗*^
Vaccine	3 (1.6)	0 (0.0)	3 (1.9)	1.000^*∗*^
Acetaminophen	7 (3.8)	1 (4.3)	6 (3.7)	1.000^*∗*^
Insect sting	1 (0.5)	0 (0.0)	1 (0.6)	1.000^*∗*^
Food	121 (65.8)	12 (52.2)	109 (67.7)	0.142
Seafood	18 (9.8)	3 (13.0)	15 (9.3)	0.476^*∗*^
Wheat	8 (4.3)	1 (4.3)	7 (4.3)	1.000^*∗*^
Buckwheat	2 (1.1)	0 (0.0)	2 (1.2)	1.000^*∗*^
Pupa	2 (1.1)	0 (0.0)	2 (1.2)	1.000^*∗*^
Nut	5 (2.7)	2 (8.7)	3 (1.9)	0.118^*∗*^
Egg	23 (12.5)	4 (17.4)	19 (11.8)	0.498^*∗*^
Kiwi	2 (1.1)	0 (0.0)	2 (1.2)	1.000^*∗*^
Pork	3 (1.6)	0 (0.0)	3 (1.9)	1.000^*∗*^
Cow milk	34 (18.5)	1 (4.3)	33 (20.5)	0.083^*∗*^
Idiopathic	30 (16.3)	1 (4.3)	29 (18.0)	0.132^*∗*^

^*∗*^Fisher's exact test. NSAID = nonsteroidal anti-inflammatory drug.

**Table 3:** Clinical characteristics of anaphylaxis cases accurately or inaccurately.

	Overall (*n* = 184)	Accurate (*n* = 23)	Inaccurate coding (*n* = 161)	*p*
Symptoms (%)				
Cutaneous	180 (97.8)	22 (95.7)	158 (98.1)	0.417^†^
Respiratory	83 (45.1)	15 (65.2)	68 (42.2)	0.038
Cardiovascular	18 (9.8)	9 (39.1)	9 (5.6)	0.001^†^
Gastrointestinal	116 (63.0)	6 (26.1)	110 (68.3)	0.001
Neurologic	7 (3.8)	3 (13.0)	4 (2.5)	0.043^†^

Blood pressure (mmHg)				
Systolic blood pressure	98.0 (95–105)^*∗*^	104.0 (95–111)^*∗*^	98.0 (95–105)^*∗*^	0.104^‡^
Diastolic blood pressure	60.5 (58–69)^*∗*^	69.0 (58–76)^*∗*^	60.0 (57–67)^*∗*^	0.095^‡^
Severe symptoms	10 (5.4)	7 (30.4)	3 (1.9)	0.001^†^
Nonalert consciousness	2 (1.1)	2 (8.7)	0 (0.0)	0.015^†^

ED treatment				
Oxygen supply	7 (3.8)	2 (8.7)	5 (3.1)	0.213^†^
Fluid administration	64 (34.8)	19 (82.6)	45 (28.0)	0.001
Antihistamine use	177 (96.2)	22 (95.7)	155 (96.3)	1.000^†^
Steroid use	51 (27.7)	14 (60.9)	37 (23.0)	0.001
Epinephrine use	36 (19.6)	15 (65.2)	21 (13.0)	0.001^†^
Bronchodilator use	27 (14.7)	7 (30.4)	20 (12.4)	0.051^†^

^*∗*^Median (interquartile range); ^†^Fisher's exact test; ^‡^Mann–Whitney test. ED = emergency department.

**Table 4:** Multivariate logistic regression analysis for factors associated with accurate anaphylaxis registration.

	Odds ratio	95% confidence interval	*p*
Cardiovascular symptom	5.872	1.529–22.548	0.010
Fluid administration in ED	4.507	1.156–17.572	0.030
Epinephrine use in ED	5.981	1.784–20.055	0.004

ED = emergency department.

**Table 5:** Administration frequency of the 3 major emergency anaphylaxis drugs.

Used medicine	Overall (*n* = 184)	Accurate (*n* = 23)	Inaccurate coding (*n* = 161)
None (%)	4 (2.2)	1 (4.3)	3 (1.9)
Antihistamine only	115 (62.5)	7 (30.4)	108 (67.1)
Epinephrine only	0 (0.0)	0 (0.0)	0 (0.0)
Steroid only	2 (1.1)	0 (0.0)	2 (1.2)
Antihistamine + epinephrine	13 (7.1)	1 (4.3)	12 (7.5)
Antihistamine + steroid	28 (15.2)	0 (0.0)	28 (17.4)
Epinephrine + steroid	1 (0.5)	0 (0.0)	1 (0.6)
Antihistamine + epinephrine + steroid	21 (11.4)	14 (60.9)	7 (4.3)

## Data Availability

The datasets generated during and/or analyzed during the current study are available from the corresponding author on reasonable request.
